# Congenital plaque-type blue nevus on the palm of the right hand: a rare case report

**DOI:** 10.3389/fped.2025.1626876

**Published:** 2026-01-07

**Authors:** Lin Qi, Guoliang Jia, Yanlong Wang, Chuanjie Xu, Chunli Yao

**Affiliations:** 1 Department of Dermatology, The Second Hospital of Jilin University, Changchun, China; 2 Department of Orthopedics, The Second Hospital of Jilin University, Changchun, China; 3 Department of Pathology, The Second Hospital of Jilin University, Changchun, China

**Keywords:** blue nevus, case report, PALM, PTBN, surgery

## Abstract

Plaque-type blue nevus (PTBN) is a rare variant of blue nevus that mostly involves the breast, head, neck, trunk, and, rarely, the hand and palm. In this study, we report a rare case of congenital PTBN involving the right palm since birth. A 10-year-old girl presented to our department with plaque in her right palm accompanied by a tingling sensation for 1 month. Histochemical analysis indicated a heavily pigmented melanocyte proliferation in the dermis, subcutis, and fascia. The patient was diagnosed with congenital PTBN and underwent surgical excision. No recurrence was observed during a 5-year follow-up.

## Introduction

Plaque-type blue nevus (PTBN), widely described as a rare variant of blue nevus (BN), appears as a blue–gray pigmented area measuring 1 cm or more in diameter (1, 2). Most patients present with congenital and asymptomatic plaques, involving the breast (1), head, neck, trunk (3), and, rarely, the hand and palm (4).

Here, we present a rare case of a PTBN occurring in the right palm and summarize the clinical course and histological characteristics in context with published reports.

## Case presentation

**Figure 1:**
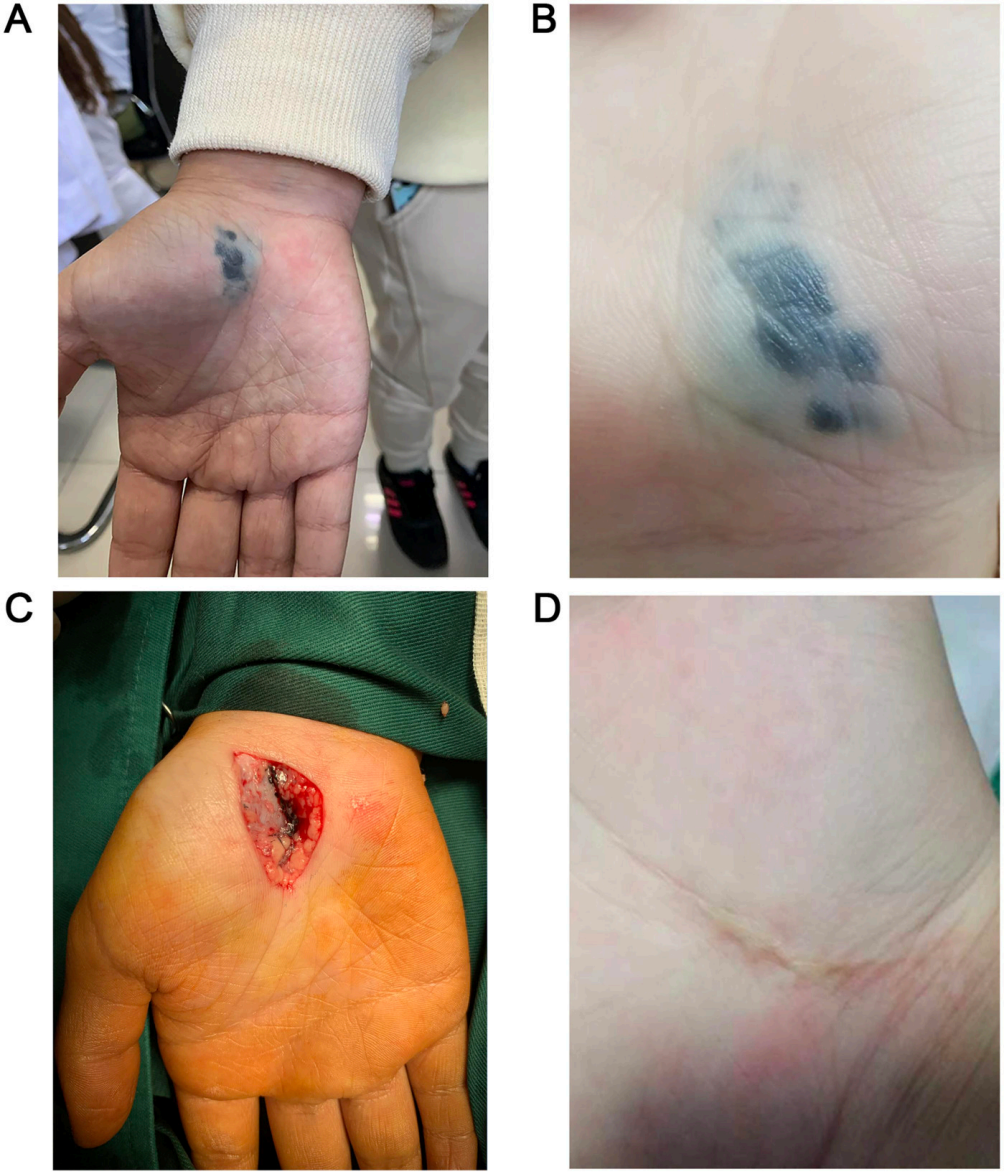
Clinical examination and intraoperative findings during excision of plaque-type blue nevus. **(A,B)** Plaque-type blue nevus of the right palm showed bluish and irregular characteristics. **(C)** Intraoperative findings presented several small black nodules. **(D)** Clinical examination suggested no evidence of recurrence during 2-year follow-up.

The reporting of this study conforms to the CARE guidelines (5). A 10-year-old girl presented to the Dermatology Department of The Second Hospital of Jilin University (Changchun, China) in November 2019 due to a plaque on her right palm accompanied by a tingling sensation for 1 month. The plaque had been present since birth and showed gradual thickening approximately 8 months before presentation. On physical examination, a blue plaque (26 mm × 12 mm) with nodular thickening was observed on the right palm, with an irregular, smooth, and localized surface that was slightly elevated (Figures 1A,B). The patient reported no family history of melanoma. The nevus appeared as a single, well-defined papule that was slightly elevated and predominantly steel blue in color (Figure 1B). Based on the patient's medical history and clinical presentation, a preliminary diagnosis of PTBN was made. Her parents were informed that typical PTBNs are benign cutaneous lesions for which close clinical observation is generally appropriate. For locally thickened nodules, histopathological confirmation via skin biopsy may be warranted. They were also advised that although most PTBNs remain benign, a subset may progress to large PTBN with subcutaneous nodules, a condition associated with a potential risk of malignant transformation. Prophylactic surgical excision was discussed as an alternative management option. After a thorough discussion with the care team and consideration of the potential malignant risk, the parents elected to proceed with surgical excision. Surgical excision was performed under local anesthesia. In addition to the blue-stained skin tissue, a large amount of blue-stained tissue was scattered between the subcutaneous fat tissue and the surface of the thenar aponeurosis, showing a pattern resembling a “splashed ink” (Figure 1C).

**Figure 2:**
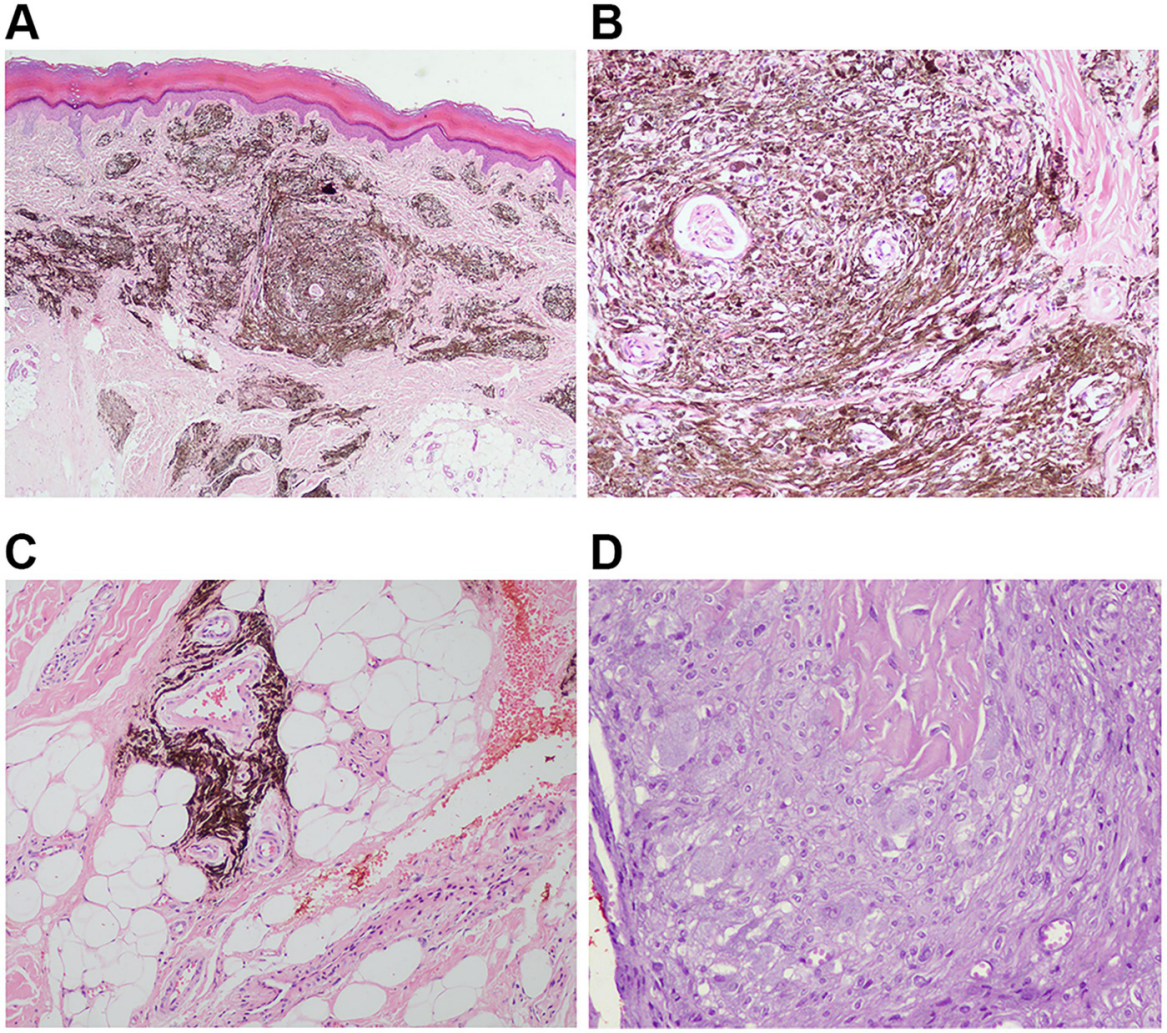
Hematoxylin–eosin (H&E) staining of skin biopsy from the right palm at 40× **(A)**, 100× **(B)**, 200× **(C)**, 200× **(D)** magnification. **(A,B)** There was hyperkeratosis of the epidermis, along with a large number of dendritic melanocytes in the superficial and middle layers of the dermis, which infiltrated the dermis and subcutaneous tissue. **(C,D)** There were multiple nested melanocytic proliferations in the subcutaneous fibrous connective tissue. The proliferative nodules are rich in cells, with enlarged nuclei. The nucleoli were locally available with no definite nuclear division phase.

**Figure 3:**
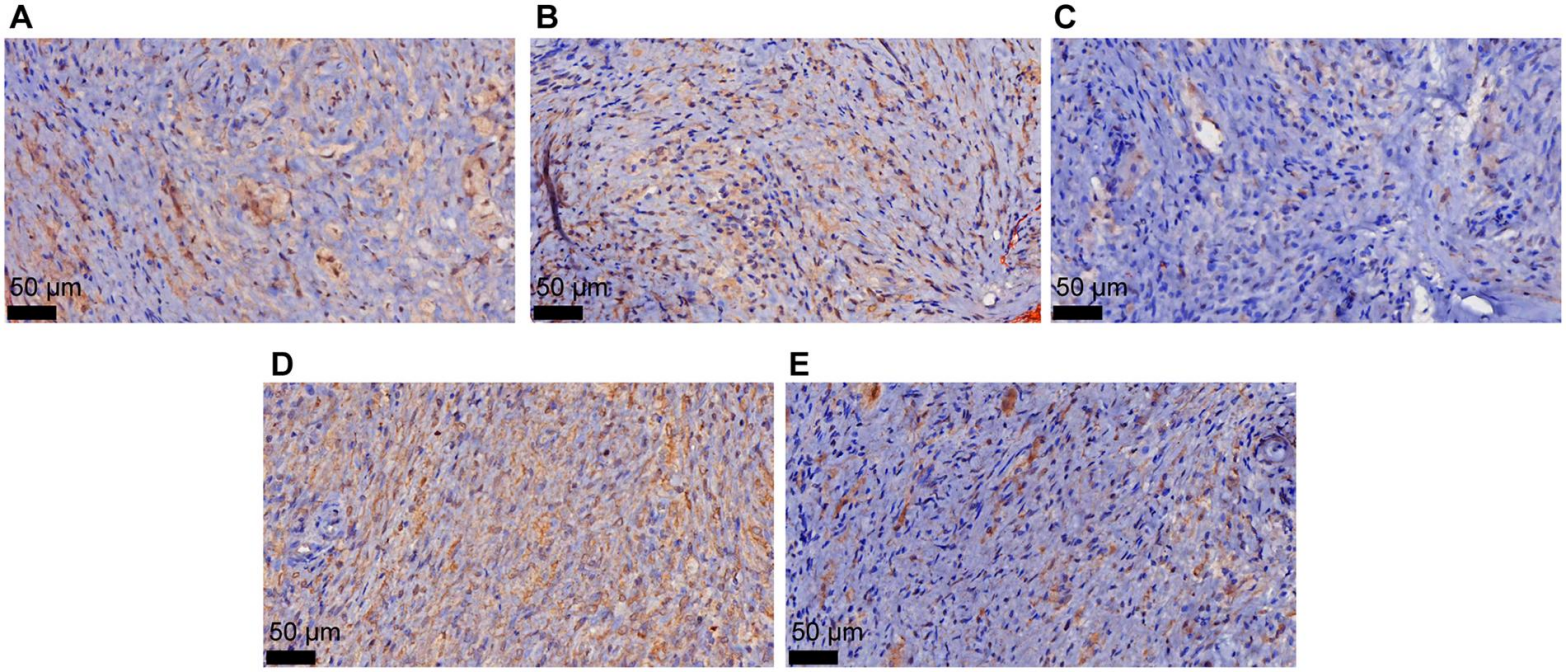
IHC staining for BAPI **(A)**, HMB45 **(B)**, Ki-67 **(C)**, MelanA **(D)**, and SOX10 **(E)**. The images were observed under a magnification of 400×.

Histopathological examination revealed epidermal hyperkeratosis, accompanied by a dense proliferation of dendritic melanocytes in the superficial and mid-dermal layers, with infiltration extending into the dermis and subcutaneous tissue (Figures 2A,B). As shown in Figures 2C and D, multiple nested melanocytic proliferations were present in the subcutaneous fibrous connective tissue. The proliferative nodules were rich in cells with enlarged nuclei, and nucleoli were locally visible, with no evidence of nuclear division. Immunohistochemical (IHC) staining revealed positivity for MelanA, HMB45, SOX10, and BAP1, and the Ki-67 positive index was <5% (Figures 3A–E).

**Figure 4:**
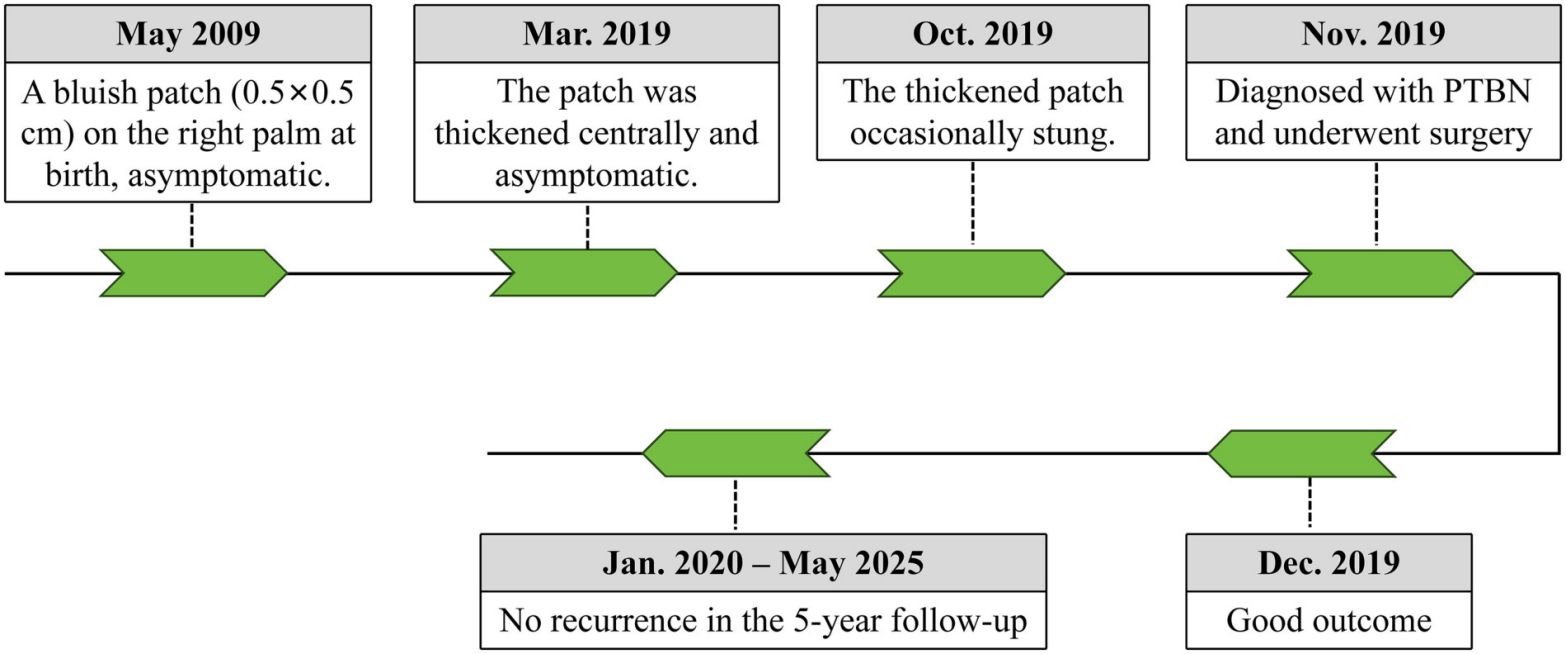
A diagram for the clinical course, interventions, and follow-up.

After differential diagnosis among common, cellular, and malignant BN, the patient was ultimately diagnosed with congenital PTBN and underwent surgical excision of the lesion. No recurrence was observed during the 5-year follow-up (Figure 1D). A diagram illustrating the clinical course, interventions, and follow-up is shown in Figure 4.

## Discussion

PTBN may be acquired, typically presenting as a solitary lesion, but it can also be congenital and manifest at multiple sites, characterized by the presence of multiple solid, slightly elevated nodules within the blue plaques. It most commonly occurs on the trunk, head, or breast, with occasional involvement of the face and mouth (6–10). Rarely, the hands and palms may be affected. In this study, we report a rare case of PTBN involving the palm.

PTBN is a relatively rare condition. Among two previously reported cases of palm PTBN (4, 7), case 1 underwent a biopsy, which revealed an ordinary BN, and the lesion was subsequently monitored through regular follow-up. The biopsy of palm case 2 (4) was also benign; however, a subcutaneous nodule on the same patient's upper arm exhibited features consistent with a cellular BN with melanoma-like characteristics, highlighting that PTBN warrants close follow-up because of its potential for malignant transformation. In addition, the clinical manifestations of the present case are similar to those of breast PTBN in the literature (1). This patient with breast PTBN underwent a total mastectomy because puncture biopsy revealed an atypical melanocytic tumor with concern for melanoma ex-blue nevus, further underscoring the malignant potential of PTBN.

Embryonic inheritance may contribute to the arrest of melanocyte migration in ectopic dermis, which in turn promotes proliferation of melanocytes within dermal tissues, leading to the development of BN (4, 11). During formation of the peripheral nervous system, migration of neural crest-derived cells (e.g., melanocytes and neuronal cells) is inhibited, resulting in the distribution of PTBN along the nerves (4, 12). Although the median nerve was not involved in this case, the lesion was not confined to the skin alone, supporting the hypothesis that PTBN is not of epithelial origin.

Clinically, PTBN may present as bluish or gray patches, plaques, or papules (6, 7, 13, 14). Most lesions are localized to the head, neck, and trunk, although some may extend into subcutaneous fat, fascia, or even skeletal muscle (15). In patients with PTBN exhibiting subcutaneous nodules, the possibility of melanoma should be considered due to its malignant potential. In this case, following thorough discussion between the care team and the patient's parents, surgical excision was chosen. Pathological analysis confirmed involvement of the subcutaneous fat and aponeurosis. Surgery was performed successfully, and no recurrence was observed during the 5-year follow-up.

Histologically, PTBN is characterized by multifocal dermal or subcutaneous proliferation of dendritic pigmented melanocytes (16). Benign melanocytes may involve subcutaneous tissue and surrounding hair follicles, nerves, and blood vessels, without cytologic heterogeneity (7). The trunk variant of PTBN often presents as large, plaque-like lesions appearing at birth or during childhood, with subcutaneous nodules developing several years later (17). Multiple nodules of cellular BN and common BN may extend into underlying soft tissues, fascia, or breast tissues (17–19). Accordingly, PTBN frequently affects deep tissues through infiltrative growth.

In clinical practice, differential diagnosis should include PTBN, BN, cellular BN (CBN), and malignant BN (MBN). Common BN is frequently encountered in routine pathological practice and usually presents as a solitary, dome-shaped papule, with a uniform blue to blue–black coloration. Histologically, it consists of a nodular or vaguely nodular proliferation of spindle-shaped melanocytes with minimal pigmentation, along with deeply pigmented dendritic melanocytes embedded within thickened collagen bundles in the mid to upper dermis (20). Cellular BN presents as an enlarged, elevated nodule or plaque, with a smooth or slightly irregular surface, commonly involving the buttocks and sacrococcygeal region, as well as the scalp and face. Histologically, the lesion is composed of fascicles or nests of tightly packed, moderately sized spindle to oval melanocytes, accompanied by scattered melanophages, forming a well-demarcated nodule (21). Malignant BN, including BN-like melanoma and BN-associated melanoma, typically shows pathological features such as increased cellular atypia, active mitotic activity, and focal necrosis, along with immunohistochemical alterations including an elevated Ki-67 proliferation index and loss of BAP1 expression. In this case, immunohistochemical staining showed positive BAP1 expression and a low Ki-67 proliferation index, suggesting a benign lesion. The patient was finally diagnosed with PTBN based on the clinical and pathological findings.

Our understanding of the molecular mechanism underlying PTBN remains limited. The predominant hypothesis is that an embryological genetic event induces a migratory arrest of melanocytes in ectopic dermal locations. Activating mutations in G proteins are relatively specific for blue nevi and related melanomas located in the skin. The most frequently observed mutations in PTBN involve *GNAQ* or *GNA11* (21). Lesions harboring *GNA11* mutations tend to arise in the upper body, particularly the scalp, whereas *GNAQ*-mutated tumors are more commonly found in the sacrum and the dorsum of the hands or feet (22).

To date, there is still consensus on the treatment regimens of PTBN (23, 24). Expanded BN involvement is one of the surgical indication in PTBN patients. In cases with lymph node involvement, excision of the primary skin lesion and lymph node biopsy are recommended, followed by histological and immunohistochemical analysis (15, 25). Surgery may also be indicated for cosmetic reasons. After radical resection, skin defects should be managed according to plastic surgery principles (26, 27). In addition, the benign or malignant potential should be carefully considered in patients with large (>2 cm) or multinodular BN, particularly on the scalp and in elderly patients with rapid or progressive changes. For PTBN, the size and location of the lesions should be assessed. Lesions unsuitable for surgical excision are recommended for close follow-up, with histopathological evaluation of any newly arising nodules. When the size and location are amenable to surgery, prophylactic excision may be performed to prevent potential malignant transformation. PTBN may respond poorly to laser therapy; however, in cases coexisting with Ota nevus, laser treatment may be selectively applied to the Ota nevus component (28). In this case, the patient was diagnosed with benign PTBN, who showed no recurrence in the 5-year follow-up after surgery.

## Conclusions

Most PTBN patients showed lesions in the trunk, head, neck, and breast, with rare cases showing palm involvement. In this study, we reported a 10-year-old girl with PTBN in the right palm. The patient showed no recurrence after surgery in the long-term follow-up as the conditions were benign.

## Data Availability

The raw data supporting the conclusions of this article will be made available by the authors, without undue reservation.
